# Intraoperative radiation therapy (IORT) in pancreatic cancer

**DOI:** 10.1186/s13014-016-0753-0

**Published:** 2017-01-19

**Authors:** Robert Krempien, Falk Roeder

**Affiliations:** 1 Department of Radiation Oncology, Helios Clinic Berlin-Buch, Schwanebecker Chaussee 50, Berlin-Buch, 13125 Berlin, Germany; 2 Department of Radiation Oncology, University Hospital of Munich (LMU), Munich, Germany; 3 Clinical Cooperation Unit Molecular Radiation Oncology, German Cancer Research Center (DKFZ), Heidelberg, Germany

## Abstract

Despite the important improvements made in the fields of surgery, chemotherapy and radiation therapy, pancreatic cancer remains one of the most lethal malignancies. Improved outcomes with novel chemotherapy regimes led again to increased attention on the role of localized radiotherapy, since local tumor progression causes significant morbidity and mortality in patients. Even after resection local failure rates are as high as 50–80%. The immediate proximity to critical structures (bone marrow, spinal cord, kidneys, liver, and intestine) limits the dose of radiation that can be administered to the tumor bed with conventional external beam radiation therapy (EBRT). The intraoperative radiotherapy (IORT) appears to be an ideal therapeutic strategy for this disease, having the advantage of enabling the delivery of high doses of radiation to areas that are at risk for microscopic disease, saving critical organs and reducing the possibility of inducing radiotoxicity. This technique allows a theoretical increase in the radiation therapeutic index to tumor compared to the adjacent organs at risk (OAR). The aim of this review is to update and comment on IORT in the multidisciplinary management of pancreatic cancer.

## Background

Pancreatic cancer is the fourth commonest cause of death from cancer in men and women [1, 2]. Surgical therapy currently offers the only potential monomodal cure for pancreatic adenocarcinoma [3]. However, only a few patients present with tumors that are amenable to resection, and even after resection of localized cancers, long-term survival is rare. At presentation, only 10–20% of patients with pancreatic adenocarcinoma have potentially resectable cancers, 40% have locally advanced unresectable tumors, and 40% have metastatic disease. Adenocarcinoma of the pancreas has a 5-year survival rate of only 4% [2]. In spite of the progress in surgical treatment, resulting in increased resection rates and a decrease in treatment-related morbidity and mortality, resection has failed to improve long-term survival [3]. By histological evaluation  <  15% of the patients undergoing R0 resection have a pN0 status, >50% suffer from lymphangiosis carcinomatosa, and  >  50% suffer from extrapancreatic nerve plexus infiltration [4, 5]. Combined modality treatment approaches using chemotherapy or chemoradiation in addition to surgery are the mainstay in treatment of pancreatic cancer [4–8].

## Achievements of surgery

Although surgery offers a low cure rate, it is also the only chance for cure. Regarding long-term survival after R0 resection, only 3–16% of the patients from selected series survived 5 years or more. Locoregional recurrence and/or metastatic disease develop in the majority of patients who undergo pancreatic resection. Relapse occurs within 9–15 months after initial presentation and patients have median life expectancies of only 12–15 months without adjuvant therapy. The 5-year survival rate of patients with resected pancreatic adenocarcinoma is approximately 10% [3]. The statistics for the 80–90% of patients who present with locally advanced and metastatic pancreatic cancer are even more dismal. Rarely do such patients achieve a complete response to treatment; median survival is 5–10 months and 5-year survival is near zero [6].

The cardinal rule in improving the prognosis in patients with pancreatic cancer proved to be complete tumor removal in patients undergoing oncological resection [3, 4]. In most recent published prospective trials, R0 resection results in an increase of survival in comparison with patients with a residual tumor [7, 8]. However, R0 resection fails to improve long-term survival [4]. More than 95% of the patients undergoing surgical resection are at an advanced stage of cancer. Potentially curative resection is hampered by a failure to include remote cancer cell-positive tissues in the operative specimen, i.e. N2 lymph nodes, nerve plexus, and perivascular tissue [9, 10]. Cancer recurrence after resection with curative intent is the consequence of cancer cell-positive tissues left behind. However, comparison of the survival times after standard and extended resection of pancreatic cancers indicated that no significant long-term survival benefit resulted from extended R0 resection [11, 12].

## Dissemination pattern of pancreatic cancer

Using molecular biological methods like reverse transcriptase polymerase chain reaction (RT-PCR) or immunostaining, a new dimension of micrometastasis has been objectified. With the higher sensitivity of these molecular biological methods, up to 60% of lymph nodes previously seen as microscopically free of cancer showed micrometastasis by RT-PCR even in UICC stage I or II cancers [5, 13]. Nerve plexus invasion outside of the pancreas has been observed in 43–72% of patients [14, 15]. Further, careful histopathological evaluation of cancer dissemination has demonstrated that even in stage I and II cancers, lymph vessels surrounding the pancreas are cancer cell infiltrated in most of the cases [5, 15].

This knowledge about cancer cell dissemination early in the course of pancreatic cancer, including early stage cancers, explains why true R0 resection in pancreatic cancer is difficult to achieve, and explains the observed frequency of recurrence in >95% of patients undergoing surgical resection with curative intent.

## Combined modality treatment

Both distant and local/regional patterns of recurrence are common, and this suggests that most patients have occult metastatic or local/regional disease (or both) at the time of resection. According to several phase II or III trials, combined modality treatment approaches using chemotherapy or chemoradiation in addition to surgery can achieve improvement in locoregional control and survival [7, 8, 16]. Novel systemic treatment regimens like FOLFIRINOX and nab-paclitaxel have demonstrated improvements in prolonging survival in advanced cases, but long-term survival is still scarce [17, 18].

## Radiation therapy dose escalation

Several trials could show that dose escalation in radiation therapy resulted in improved local control in combination with potentially curative resection [19–21]. The efficacy of external beam radiation therapy (EBRT) in pancreatic cancer is limited by the inability to deliver adequate doses of irradiation secondary to the dose tolerance limits of small bowel, spinal cord, stomach, kidney, and liver. Further, the use of combined modality approaches in pancreatic cancer is associated with increased gastrointestinal toxicity. Technical developments like Intraoperative Radiation Therapy (IORT) have the potential to significantly improve radiation therapy of pancreatic cancers by reducing normal tissue dose, and simultaneously allow for escalation of dose to further enhance locoregional control [21, 22].

## Intraoperative Radiation

Intraoperative radiation therapy (IORT) techniques allow for the delivery of high doses of radiation therapy while excluding part or all of the nearby dose-limiting sensitive structures. Therefore, the effective radiation dose is increased and local tumor control potentially improved. This is pertinent in the case of pancreatic cancer because local failure rates are as high as 50–80% in patients with resected and locally advanced disease [10]. In pancreatic cancer, IORT has been offered for unresectable tumors to provide local tumor control and palliation of pain, and for resectable tumors in an effort to improve local control and survival after PD [21].

### Technique of IORT

**Fig. 1:**
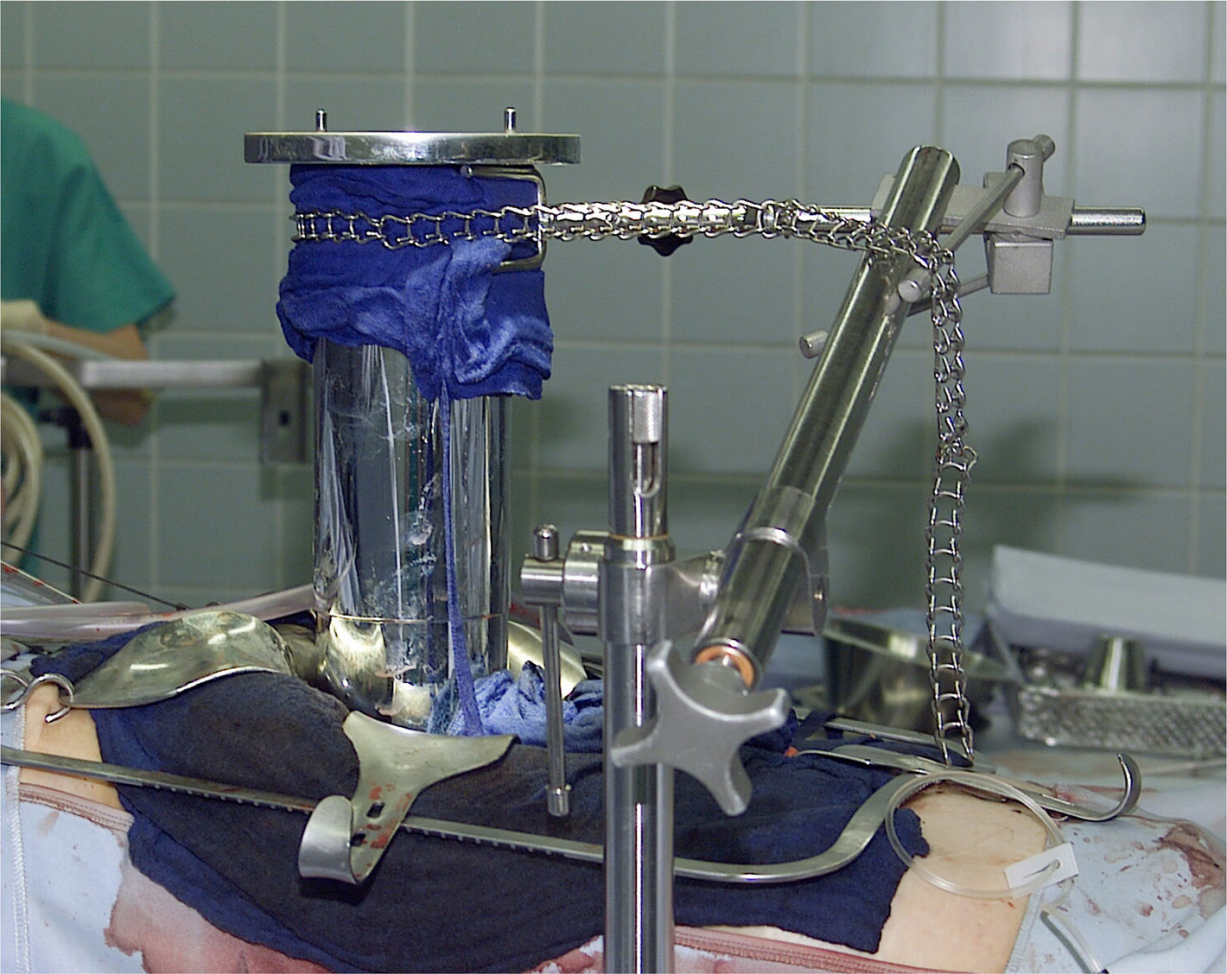
Intraoperative radiation Therapy with electrons (IOERT). Placement of an intraoperative electron beam applicator in a patient with pancreatic cancer. An applicator of appropriate size is chosen, manually positioned and attached to the table. Applicators are made of steel or plastic to restrict the radiation field laterally and are usually available in different sizes, shapes and bevel angles. Prior to irradiation, the axis of the applicator has to be aligned properly with axis of the LINAC in a defined distance. The dose is usually prescribed to the 90% isodose

**Fig. 2:**
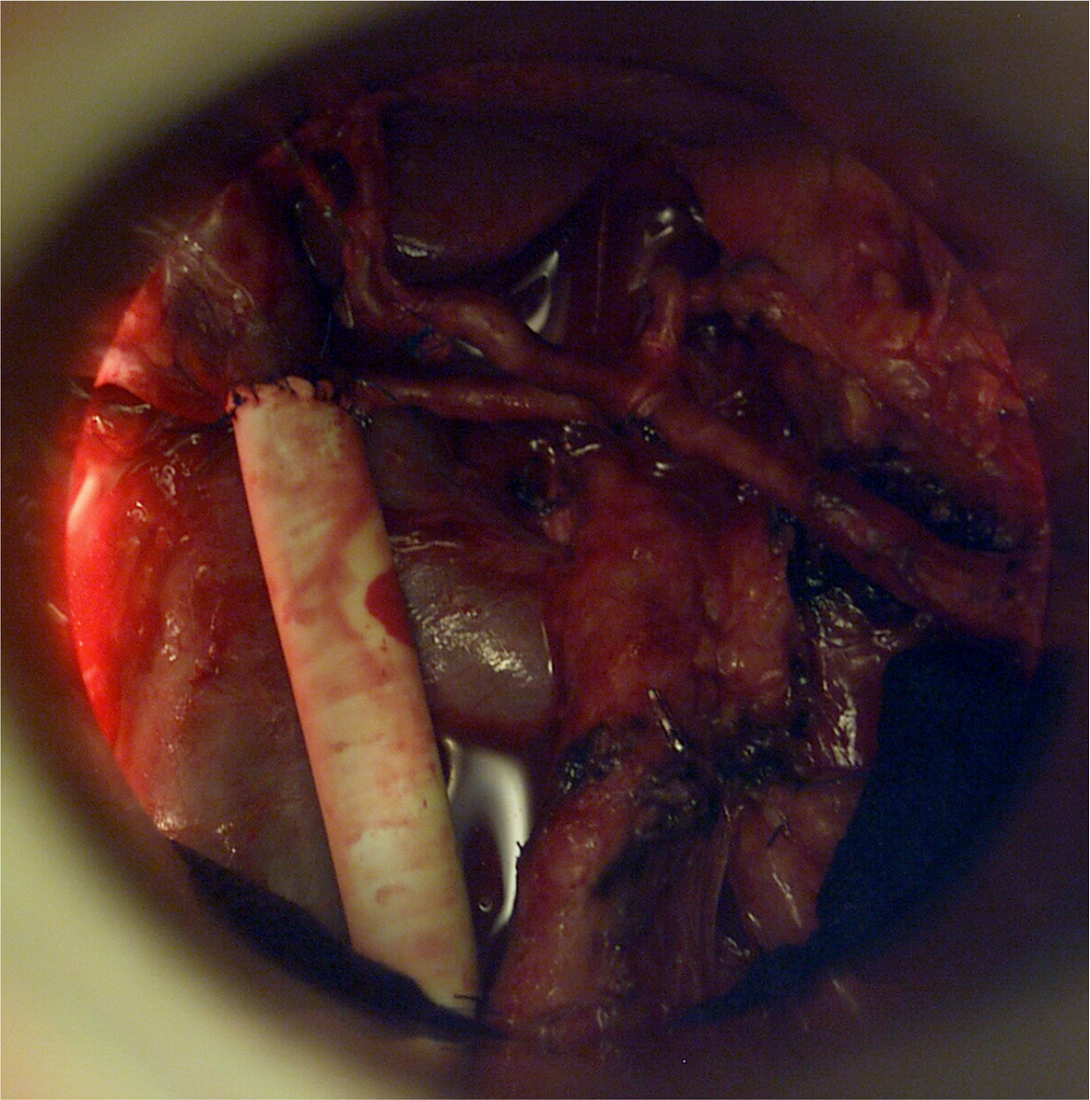
Beams eye view though a intraoperative electron beam applicator to the tumor bed after resection of a locally advanced pancreatic cancer

Intraoperative radiation therapy is defined as the application of a single fraction of high dose irradiation during a surgical procedure. The target volume is usually the tumor bed after gross total resection or the remaining gross residual disease if resection was not (completely) possible. IORT is typically used as a boost after preoperative or prior to postoperative EBRT. The use of IORT alone should be restricted to situations without a reasonable opportunity for EBRT (for example recurrence after prior irradiation). Technically, two major approaches are in use for IORT treatments of pancreatic cancer: electrons and HDR-brachytherapy [23, 24]. After surgical removal of the tumor, the target volume is defined by the radiation oncologist in correspondence with the treating surgeon (Figs. 1 and 2). An IORT boost offers several advantages compared to an EBRT boost: first of all, radiosensitive structures or organs at risk can be surgically moved out of the radiation field and therefore effectively spared from radiation exposure. Target volume definition takes place under visual control which minimizes a possible geographical miss. Safety margins can be kept to a minimum as no substantial intra- or interfractional movements have to be taken into account and finally overall treatment time is shortened. These advantages have to be weighed against some drawbacks: No final pathological assessment of margin status will usually be available for treatment stratification. Late toxicity might be increased at least theoretically due to the use of a high single dose. Three-dimensional treatment planning is not (yet) available, treatment documentation can be difficult and finally doing IORT is a major interdisciplinary effort and therefore only available at large centers [23].

Typically single doses of 10–20 Gy are used. However, the conversion of high single doses into biologic equivalent doses in fractionated therapy is difficult. For example, a single dose of 15 Gy would be equivalent to 31–54 Gy in conventionally fractionated RT regarding tumor and late reacting tissue response using the linear-quadratic model and assuming alpha/beta values of 3–10 [18, 19]. However the linear quadratic model is not validated for high single doses and probably overestimates the equivalent fractionated dose [25, 26]. Further on there is growing evidence for a different tissue reaction to high single doses per se if a threshold of 8–10 Gy is exceeded [27]. Taken alternative models [26] and the clinical experience into account, it seems more reliable to assume an equivalent fractionated dose which is 2–3 fold the IORT dose, while the tumor effect seems rather 2 fold and the late reacting tissue effect rather 3-fold. This underlines the need for optimal sparing of organs at risk and the combination of IORT with EBRT whenever feasible.

## IORT in pancreatic cancer: The Beginning

Initial studies were conducted at the University of Kyoto. In these early studies resection was followed by IORT doses of 25–30 Gy [28]. Nishimura et al. reported an improvement in survival and pain relief in a series of patients with advanced pancreatic cancers treated with IORT doses of 20–40 Gy compared with the control arm [29]. These studies prompted further investigations. Shipley et al. evaluated 20 Gy IORT in patients with unresectable disease in combination with EBRT. Median survival was 16.5 month with approximately 50% of patients achieving pain relief [30]. Roldan et al. retrospectively evaluated 159 patients treated between 1974 and 1985 with IORT in addition to postoperative EBRT. Local control was improved at both 1- and 2-year time points, though not overall survival [31].

### Safety of IORT in pancreatic cancer

Importantly, the addition of IORT after surgery did not increase perioperative complication rates significantly. Although late complications have been reported after IORT for pancreatic cancer, all reports suggest that IORT may be delivered safely even in combination with surgical resection (Tables 1 and 2) [20]. Selection of radiation doses for IORT was influenced by seminal, preclinical canine experiments performed at the National Cancer Institute that provided an understanding of normal tissue tolerances, including surgically manipulated tissues, for IORT [21, 32]. These studies created a foundation for the rational delivery of IORT in humans, so it should not be surprising that clinical studies have shown these RT doses to be safe and feasible.

**Table 1:** Selected studies of Intraoperative Radiotherapy in resected pancreatic cancer

Studies	Number	IORT Dose (Gy)	EBRT	Operative mortality	Peri-/ postoperative complications	Local recurrence	Overall survival (Median)
Sindelar et al. [33]	24	20	100%				
	12 Surgery and EBRT					100%	12 month
	12 Surgery and IORT or EBRT					33%	18 month
Zerbi et al. [34]	90	12.5–20	36%				
	47 Surgery			2.1%	23.4%	56,4%	12 month
	43 Surgery and IORT			2.3%	23.2%	26%	19 month
Alfieri et al. [35]	46	10	67%				
	20 Surgery			8%	43%	71.2	10.8 month
	26 Surgery and IORT			9%	57%	41.6%	14.3 month
Reni et al. [36]	203	10–25	28%			Median time to	
	76 Surgery			4%	45%	local failure	12 month
	127 Surgery and IORT			5%	39%	11 month 14 month	15.5 month
Ogawa et al. [37]	210	30%	63%			16.3%	19.1 month
	All patients Surgery and IORT						
Valentini et al. [38]	270 All patients surgery and IORT	7.5–25	63%			Median time to local failure 15 month	19 month
Showalter et al. [47]	99	10–20					
	46 Surgery + − EBRT		66%		40%	39%	19.2 month
	37 surgery IORT + − EBRT		74%		46%	21%	21 month

**Table 2:** Selected studies of Intraoperative Radiotherapy in locally advanced/unresectable pancreatic cancer

Studies	Number	IORT Dose (Gy)	Local Recurrence	Overall Survival (Median)
Roldan et al. [31]	159	20		
	122 EBRT		52%	12.6 month
	37 EBRT and IORT		18%	13.4 month
Tepper et al. [39]	51 EBRT and IORT	20	Not assessable	9 month
Willet et al. [40] Cai et al. [41]	194 EBRT and IORT	15–25	2 y 59%	12 month
Mohiddin et al. [48]	49 EBRT and IORT	10–20	29%	16 month
Schuricht et al. [49]	105 76 EBRT and IORT 29 EBRT	15–20	30%	15–20 month
Keane et al. [45]	85 (locally advanced/Borderline resectable)	10–20		
	26 IORT			20.5 month
	49 resected			
	24 surgery and IORT			35.1 month
	25 surgery			301.1 month

## IORT in resectable disease

In a prospective, randomized trial conducted at the National Cancer Institute (NCI) (Sindelar et al.) 24 patients were randomized to receive IORT (20Gy) with EBRT (satges II-IV). Excluding 7 perioperative deaths, an improvement in local control and median survival was seen in the patients who received IORT (OS 18 vs 12 month; *p* = 0.01) [33].

All other data regarding IORT in resectable disease are limited to retrospective single- or multi-institutional series (Table 1). Of those several have evaluated outcomes in patients who have undergone resection for pancreatic cancer, comparing cohorts who received IORT versus no IORT. Nearly all of them show a benefit due to reduced locoregional recurrence by the addition of IORT around 40–80% (Table 1).

Zerbi et all reported 90 patients who underwent resection between 1985 and 1993. 43 patients received IORT in addition to surgical resection. IORT doses were between 12.5 and 20 Gy. Results revealed similar tumorsize and overall stage between both groups and no difference in operative mortality and early postoperative complications. Local recurrence in the IORT group was 27% compared to 56% in the surgery only group whereas there was no statistically significant difference in overall or disease free survival [34]. Alfieri et al. evaluated 46 patients, of which the last 26 received IORT and adjuvant EBRT. Medium survival was 10.8 month in the surgery group and 14.4 month in the surgery and IORT group (*p* = 0.06). The 5-year local control was significantly better in the IORT group with 30% vs. 58% (*p* < 0.01). Multivariate analysis demonstrated that IORT was an independent prognostic factor for local control [35]. Reni et al. evaluated a larger series with 127 patients receiving IORT after surgery and compared these with a cohort of 76 patients with surgery alone. As in the other studies no additional operative morbidity and mortality was observed due to the addition of IORT. In Stage I and II disease IORT significantly prolonged time to local failure, time to failure and overall survival [36]. Additional data from 2 larger multi-institutional series confirmed the favourable effect of IORT on local control [37, 38].

These studies are summarized in Table 1 and suggest an improvement in local control due to IORT without additional operative morbidity or mortality.

## IORT in locally advanced or unresectable disease

In locally advanced pancreatic cancer (LAPC) the role of IORT is more clearly defined. Many studies have documented both safety and pain control with IORT, resulting in complete pain resolution in 75–90% of cases (Table 2) [20]. Nishimura et al. were among the first to show that the addition of IORT in advanced pancreatic cancer lead to a significant improvement in pain relief compared to a control group [29]. A study from Mayo Clinic evaluated 159 patients with unresectable pancreatic cancer who underwent exploratory laparotomy. Of these 37 received EBRT in combination with IORT boost. The addition of IORT to EBRT resulted in a 1-year local control of 82% compared to 48% with EBRT alone. Despite this benefit there was no difference in median or long-term survival [31].

Radiation Therapy Oncology Group (RTOG) tried to evaluate IORT in LAPC in a multi-institutional study. RTOG 8505 evaluated the role of IORT in addition to EBRT for patients with LAPC. The rate of major postoperative complications was 12% and median survival was 9 month. Local control could not adequately be evaluated in this study. This Study demonstrated the feasibility of IORT, but did not clearly show an advantage of IORT over conventional therapy [39].

Larger single institutional series have published long-term results with the use of IORT. Since 1978, patients with LAPC with good performance status have been considered for consolidative intraoperative radiotherapy (IORT) at the Massachusetts General Hospital (MGH) [4]. In 2005, Willett et al. reported that among the first 150 patients with LAPC to receive IORT at MGH as part of their treatment, the 1-year, 2-year, and 3-year overall survival (OS) rates were 54, 15%, and 7%, respectively. It is worth noting that 5 patients survived past 5 years [40]. In an updated publications of 194 consecutive patients with unresectable LAPC comparable to previously published values were reported with 1-year, 2-year, 3-year, and 5-year OS rates of 49, 16, 6, and 3%, respectively [8, 9]. Multivariate analysis showed that low comorbidity index and chemotherapy predicted improved overall survival, with a median OS of 21.2 months and a 3-year OS rate of 20% in this subgroup [41].

Novel systemic treatment regimens like FOLFIRINOX and nab-paclitaxel have demonstrated improvements in prolonging survival also in advanced cases [17, 18]. The strength of chemotherapy as a positive prognostic factor for survival in current studies supports the emphasis on upfront systemic treatment of patients with pancreatic cancer, but long-term survival is still scarce, whereas the role of radiation therapy remains controversial [7, 8].

The 2009 an autopsy analysis by Iacobuzio-Donahue et al. demonstrated that local tumor progression causes significant morbidity and mortality in patients with unresectable and even metastatic pancreatic cancer [42] leading again to increased attention on the role of localized treatment, i.e. localized radiotherapy.

Improved outcomes with novel chemotherapy regimes in the treatment of metastatic pancreatic cancer have prompted incorporation of these regimens into neoadjuvant treatment [43, 44]. While some patients are still found to be unresectable after neoadjuvant treatment, others are able to undergo resection [44]. In 2016 Massachusetts General Hospital (MGH) reported retrospectively analyzed records of 85 patients with locally advanced/ borderline resectable PDAC who received neoadjuvant treatment with chemotherapy and/or chemoradiotherapy followed by surgical exploration in an IORT-equipped operating suite between 2010 and 2015. Of 85 patients, 49 (57.6%) underwent resection after neoadjuvant treatment, 27 (31.8%) were unresectable, and 9 (10.6%) were found to have distant metastases. 24 of 49 patients who underwent resection received IORT for close/positive margins on intraoperative frozen section. There was no significant difference in operative times, postoperative complications, or operative morbidity in patients who underwent resection and IORT vs those who underwent resection alone. Median OS was 31.1 months in patients who underwent resection alone and 35.1 months in patients who underwent resection and IORT. Despite the increased incidence of close/ positive margins in patients who underwent resection and IORT, the rates of local recurrence were similar to those who underwent resection alone. 26 of 27 patients with unresectable disease upon exploration received IORT. Median OS was 20.5 months [45].

These studies are summarized in Table 2. IORT in patients with unresectable pancreatic cancer show that most patients experience pain relief and improved local control.

## IORT in locally recurrent disease

A retrospective study from the University of Heidelberg reported 36 patients with isolated local recurrences of pancreatic cancer who have been treated with a combination of surgery, IORT and EBRT. Median time from initial treatment to recurrence was 20 months. All patients were surgically explored. In 18 patients a gross total resection was achieved, whereas the other half received only debulking or no resection at all. All patients received IORT with a median dose of 15 Gy. Additional EBRT was applied to 31 patients with a median dose of 45 Gy, combined with concurrent, mainly gemcitabine-based chemotherapy. Local progression was found in 6 patients after a median time of 17 months, resulting in estimated 1- and 2-year local control rates of 91 and 67%, respectively. Distant failure was observed in 23 patients, mainly in liver or peritoneal space. The median estimated progression-free survival was 9 months with 1- and 2-year rates of 40 and 26%, respectively. They found an encouraging estimated median overall survival of 19 months, transferring into 1- and 2-year rates of 66 and 45%. Notably 6 of 36 patients (17%) lived for more than 3 years. Severe postoperative complications were found in 3 and chemoradiation-related grade III toxicity in 6 patients. No severe IORT related toxicity was observed. Combination of surgery, IORT and EBRT in patients with isolated local recurrences of pancreatic cancer resulted in encouraging local control and overall survival with acceptable toxicity [46]. This approach seems to be superior to palliative chemotherapy or chemoradiation alone and should be considered in an otherwise difficult to treat group of patients.

## Conclusion

In summary, the available data demonstrates that IORT is a safe addition to the treatment of pancreatic cancer and standard neoadjuvant or adjuvant therapies, with the intention of improving local control for patients with resectable pancreatic cancer. Series of patients with unresectable pancreatic cancer show that most patients experience pain relief and improved local control. In select studies inclusion of IORT led to improved survival. Improved outcomes with novel chemotherapy regimens led again to increased attention on the role of localized radiotherapy, since local tumor progression causes significant morbidity and mortality in patients. Intraoperative radiation therapy (IORT) techniques allow increasing the effective radiation dose and improving local tumor control. However, at present, no phase III data clearly defines the role of IORT in the management of pancreatic cancer.
